# Shedding of Marburg Virus in Naturally Infected Egyptian Rousette Bats, South Africa, 2017

**DOI:** 10.3201/eid2612.202108

**Published:** 2020-12

**Authors:** Janusz T. Pawęska, Nadia Storm, Wanda Markotter, Nicholas Di Paola, Michael R. Wiley, Gustavo Palacios, Petrus Jansen van Vuren

**Affiliations:** National Institute for Communicable Diseases of the National Health Laboratory Service, Johannesburg, South Africa (J.T. Pawęska, N. Storm, P. Jansen van Vuren);; Boston University, Boston, Massachusetts, USA (N. Storm);; University of Pretoria, Pretoria, South Africa (J.T. Pawęska, W. Markotter);; University of Nebraska Medical Center, Omaha, Nebraska, USA (N. Di Paola, M.R. Wiley, G. Palacios);; US Army Medical Research Institute of Infectious Diseases, Frederick, Maryland, USA (N. Di Paola, M.R. Wiley, G. Palacios);; Australian Centre for Disease Preparedness, Commonwealth Scientific and Industrial Research Organisation–Health and Biosecurity, Geelong, Victoria, Australia (P. Jansen van Vuren)

**Keywords:** Marburg virus, shedding, fruit bats, South Africa, viruses, zoonoses, Egyptian rousette bats, filoviruses, Rousettus aegyptiacus

## Abstract

We detected Marburg virus RNA in rectal swab samples from Egyptian rousette bats in South Africa in 2017. This finding signifies that fecal contamination of natural bat habitats is a potential source of infection for humans. Identified genetic sequences are closely related to Ravn virus, implying wider distribution of Marburg virus in Africa.

The genus *Marburgvirus*, family *Filoviridae*, comprises 1 species, *Marburg marburgvirus*, which comprises 2 marburgviruses, Marburg virus (MARV) and Ravn virus (RAVV) (*1*). Marburgviruses cause sporadic but often fatal MARV disease in humans and nonhuman primates (*2*). The Egyptian rousette bat (*Rousettus aegyptiacus*) has been implicated as the primary reservoir for marburgviruses (*3*–*9*), but the mechanisms by which they are maintained in these bats remain elusive. Evidence of marburgvirus circulation was reported from countries where MARV disease outbreaks have not been recorded (*10*–*12*). Determining the risks for spread and developing evidence-based public health strategies to prevent zoonotic transmission requires up-to-date knowledge about marburgvirus geographic range; genetic diversity; and transmission mechanisms, including natural ports of entry and shedding patterns. To clarify which marburgviruses are circulating and how they are maintained in Egyptian rousette bat populations in South Africa, we tested oral and rectal swab samples and blood samples collected during a previously identified peak season of marburgvirus transmission in a local Egyptian rousette bat population (*13*).

## The Study

We conducted this work in accordance with approved protocols by animal ethics committees of the National Health Laboratory Service (Johannesburg, South Africa; AEC 137/12) and the University of Pretoria (Pretoria, South Africa; EC054–14). During February–November 2017, a total of 1,674 Egyptian rousette bats (February, 107 bats; April, 600; May, 563; September, 214; November, 190) were captured, aged, and sampled at Matlapitsi Cave, Limpopo Province, South Africa, as described previously (*13*). We collected and processed oral and rectal swab samples from each bat and blood from a subset of 423 bats, as described previously (*7*). Swab samples were collected and pooled by aliquoting 4 × 25 μL of the media of each sample into a microcentrifuge tube, yielding a total of 416 pools containing 100 μL of pooled rectal swab samples and 4 pools containing 50 or 75 μL of pooled oral swab samples. We conducted serologic, virologic, and molecular tests as described previously (*7*). In addition, we conducted real-time quantitative reverse transcription PCR (qRT-PCR) for individual swab samples when the pool tested positive. We prepared sequencing libraries using the TruSeq RNA Access Kit (Illumina, https://www.illumina.com) with MARV-specific bait enrichment, followed by sequencing on an Illumina NextSeq, genomic alignment and phylogenetic analysis (*13*). We calculated the significance of differences in several positive swab samples and seropositivity using the Fisher exact test in Stata version 13 (StataCorp, https://www.stata.com).

Seven rectal swab pools (5 from April 2017 and 2 from September 2017) were qRT-PCR positive; the remaining rectal swab samples collected during February–November 2017 were all negative. The number of individual positive rectal swab samples ranged from 1 to 3 per positive pool, totaling 11 positive samples. Only 1 oral swab sample pool, from April 2017, yielded a positive qRT-PCR result, containing a single positive oral swab sample (Table 1). Of 600 rectal swab samples collected during 3 nights in April, 9 (1.5%) were positive; of 215 rectal swab samples collected during 2 nights in September, 2 (0.9%) were positive. We found no significant difference between the number of positive rectal swab samples collected in April and the number collected September. The number of positive rectal swab samples differed significantly from the number of positive oral swab samples collected in April (p = 0.02). Attempts to culture marburgvirus from qRT-PCR–positive swab samples were unsuccessful. Identical results from specimens with cycle threshold (C_t_) >30 were obtained in other studies (*6*,*13*).

**Table 1:** qRT-PCR results of oral and rectal swab samples from juvenile Egyptian rousette bats (*Rousettus aegyptiacus*) at Matlapitsi Cave, Limpopo Province, South Africa, February–November 2017*

Bat ID	Capture date	Sex	Swab sample type and qRT-PCR result, C_t_†	
			Oral	Rectal
Apr-77	Apr 4	F	Negative	30.61
Apr-117	Apr 4	F	Negative	30.57
Apr-118	Apr 4	F	Negative	30.95
Apr-124	Apr 4	F	Negative	30.66
Apr-133	Apr 4	F	Negative	30.46
Apr-232	Apr 5	F	33.01	Negative
Apr-369	Apr 6	F	Negative	31.9
Apr-380	Apr 6	M	Negative	32.67
Apr-399	Apr 6	F	Negative	31.7
Apr-470	Apr 6	M	Negative	31.8
8025	Sep 26	F	Negative	31.7
0895	Sep 20	F	Negative	33.05

*C_t_, cycle threshold; ID, identification; qRT-PCR, quantitative real-time reverse transcription PCR. †Negative, C_t_ >40.

We obtained sufficient marburgvirus-specific sequence data only from 1 of the 12 individual positive swab samples for phylogenetic analysis: a rectal swab sample, collected from a juvenile female (bat 8095) in September 2017, from which we recovered 79.2% (15.1/19.1 kb) of the genome. We merged sequencing reads from replicate sequencing runs and mapped 2,472 reads to the MARV reference genome. Maximum coverage per base obtained was 291 reads; some regions had no coverage. The average coverage per base across the genome was 18.5 reads (when we included 0 coverage regions), and the average coverage when we excluded 0 coverage regions was 40 reads. We obtained near-complete coding sequences for the viral protein (VP) 35 (972/990 nt; 98.2%) and VP40 (898/912 nt; 98.5%) genes; coverage ranged from 49.7% (VP24) to 89.3% (glycoprotein) in other open reading frames of the genome.

**Figure 1:**
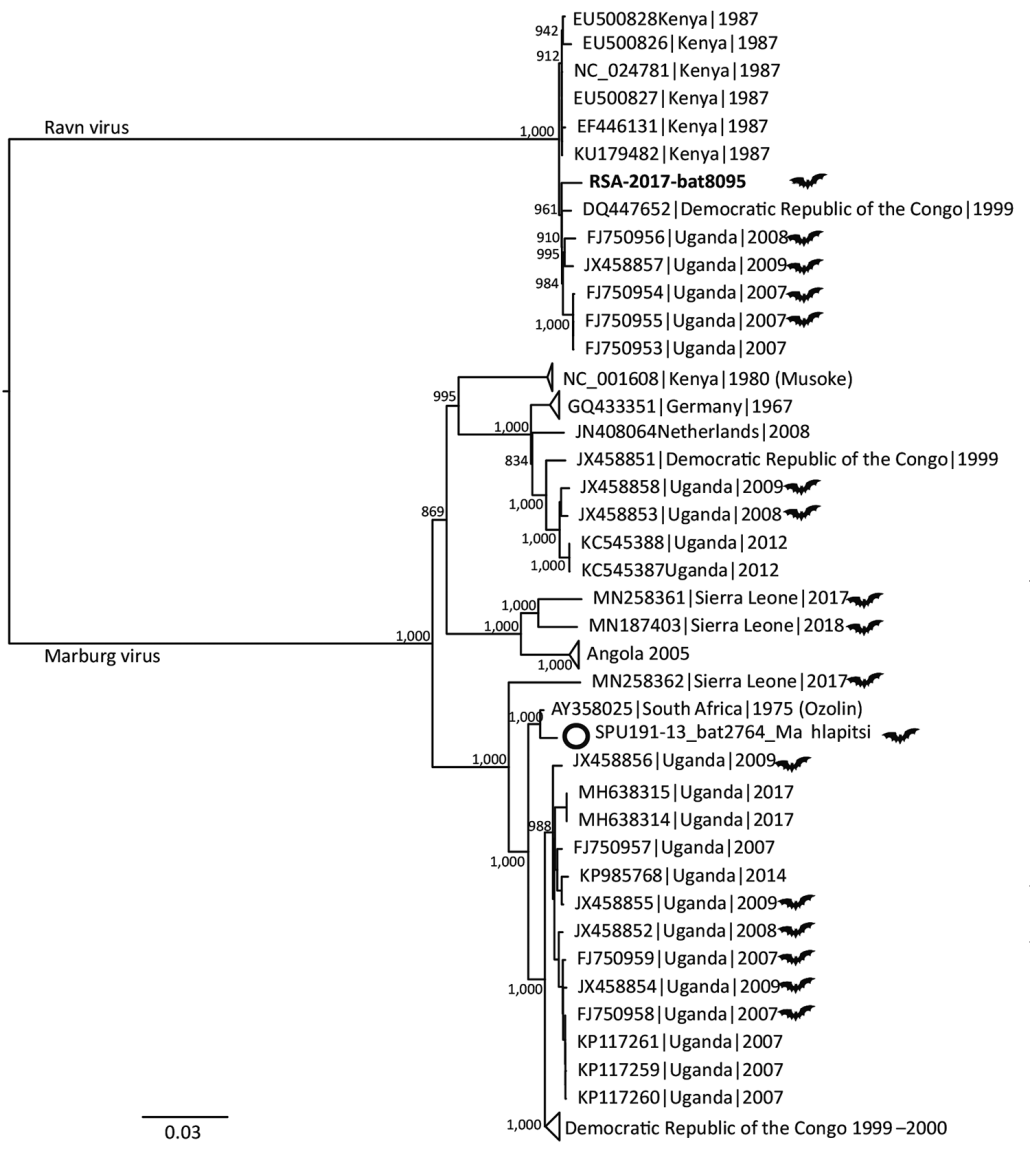
Midpoint-rooted, maximum-likelihood phylogeny of complete and near-complete MARV and RAVV genomes. Phylogenetic tree shows evolutionary relationships of marburgvirus detected in a rectal swab sample from a subadult Egyptian rousette female bat (*Rousettus aegyptiacus*) in Matlapisi Cave, Limpopo Province, South Africa, 2017 (black filled circle; GenBank accession no. MT321489), and reference viruses, including the SPU191-13 bat 2764 Mahlapitsi strain (white circle; GenBank accession no. MG725616), detected in the same cave in July 2013. Complete and near-complete genome sequences from GenBank (accession numbers indicated) were aligned with the partial MARV sequence obtained from RSA-8095bat using MUSCLE version 3.8.31 (https://www.drive5.com/muscle), and RAxML version 8.2.10 (https://cme.h-its.org/exelixis/web/software/raxml/index.html) was used to infer the best-scoring maximum-likelihood tree after 1,000 bootstrap replicates. Node values indicate the bootstrap support values. Genomes isolated from bats are shown using a bat symbol. Scale bar indicates nucleotide substitutions per site. MARV, Marburg virus; RAVV, Ravn virus.

The marburgviruses sequence (strain RSA-2017-bat8095) detected from the rectal swab sample of bat 8095 shared a common ancestor with all other RAVV complete or near-complete genome sequences, including 3 human isolates from Kenya (*14*), Uganda (*15*), and the Democratic Republic of Congo (*3*) and several bat isolates from Uganda (*4*) (Figure 1). The RSA-2017-bat8095 nt sequence shared »77% identity with the MARV SPU191–13bat2764 Mahlapitsi strain (GenBank accession no. MG725616) that was collected and characterized from Matlapitsi Cave 4 years earlier.

**Figure 2:**
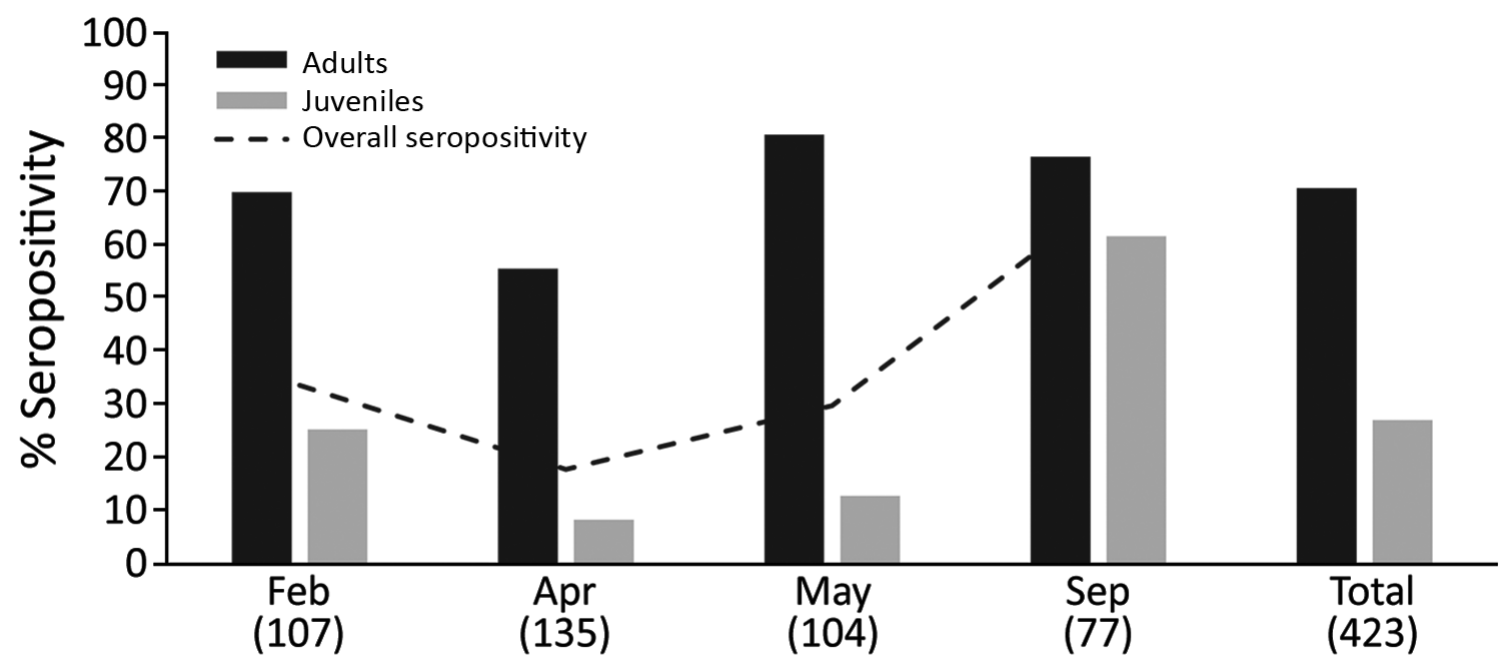
Marburgvirus seropositivity in adult and juvenile Egyptian rousette bats (*Rousettus aegyptiacus*), Matlapitsi Cave, Limpopo Province, South Africa, February–September 2017. Numbers in parentheses indicate the numbers of bats tested per month. Juvenile bats represent the new generation of bats born mostly during November 2016–January 2017.

Of 423 bats tested, 143 (33.8%) were positive for antibodies against marburgviruses (73 adults and 70 juveniles). Lowest overall seroprevalence occurred in April 2017 (17.78%) and ranged from 8.3% in juveniles (9/108) to 55.6% in adults (15/27) (Figure 2). Overall seropositivity did not differ significantly between male and female bats, but the overall seropositivity differed significantly between juvenile (forearm <89 mm; <1-year-old) and adult bats (p = 0.03). We detected seroconversion in 6 (33.3%) of 18 recaptured bats (Table 2).

**Table 2:** Marburgvirus seroconversion in 6 of 18 Egyptian rousette bats (*Rousettus aegyptiacus*) recaptured at Matlapitsi Cave, Limpopo Province, South Africa, February 2017–September 2017*

Bat ID	First capture			Recapture	
	iELISA, %†	Capture date		iELISA, %†	Capture date
SMB676	2.2	2016 Apr		23.3	2017 Feb
SMB797	12.4	2016 Jun		23.7	2017 Apr
SMA780	0.7	2014 Jul		61.7	2017 Sep
SM906	15.2	2013 Sep		202.4	2017 Sep
SMB160	2.5	2015 Mar		51.3	2017 Sep

SMB978

11.1

2016 Nov

142.2

2017 Sep

*Recaptured bats were identified by a previously applied unique tattoo number. iELISA, indirect ELISA. †Percent positivity of the internal positive control serum in I-ELISA calculated as (average optical density of the test serum replicates/average optical density of the positive control serum replicates) × 100; cutoff percent positivity of iELISA = 16.78% (7).

## Conclusions

The period of the lowest seropositivity in juveniles (April–May) resulting in the highest number of potentially susceptible bats at Matlapitsi Cave was the same as previously identified (*13*). This finding coincides with demonstrable seroconversions and virus shedding and represents a period of increased exposure. The significantly higher number of marburgvirus-positive rectal than oral swab samples we detected contrasts with results from experimentally infected Egyptian rousette bats and field studies in Uganda (*4*,*8*,*9*). Experimental data on marburgvirus shedding were obtained from subcutaneously inoculated and colonized Egyptian rousette bats (*7*–*9*). Whether this mode of infection represents a natural portal of entry for marburgviruses in Egyptian rousette bats and to what extent viral shedding patterns in colonized bats can be extrapolated to natural settings are unknown. 

Our findings highlight the risk for marburgvirus fecal environmental contamination and for Egyptian rousette bat roosting sites as a possible source of virus spillover. Roosting behavior enabling direct physical contact suggests that fecal–oral transmission of marburgviruses in bats can occur. Biting among animals or biting by hematophagous ectoparasites might result in inoculation of wounds with contaminated feces or exposure to contaminated fomites.

Our findings, combined with earlier detection of an Ozolin-like MARV in Egyptian rousette bats roosting at Matlapitsi Cave (*13*), suggest local co-circulation of multiple marburgviruses genetic variants. Detection of RAVV in South Africa, closely related to East African isolates, indicates that long-distance movement of Egyptian rousette bats contributes to widespread geographic dispersion of marburgviruses. Moreover, it implies that more virulent strains, such as the MARV Angolan strain (*2*), might be co-circulating. Entering caves and mining have been associated with MARV spillover (*3*–*6*,*14*,*15*) and detection of viral RNA in rectal swab samples, highlight the potential route of transmission. Confirmation of the period for the highest virus exposure risk further highlights the value of biosurveillance and demonstrates that marburgviruses continue endemic circulation in South Africa. This circulation represents a potential threat that needs to be communicated to at-risk communities as a part of evidence-based public health education and prevention of pathogen spillover.
